# Conformational dynamics of bacterial trigger factor in apo and ribosome-bound states

**DOI:** 10.1371/journal.pone.0176262

**Published:** 2017-04-24

**Authors:** Mehmet Tarik Can, Zeynep Kurkcuoglu, Gokce Ezeroglu, Arzu Uyar, Ozge Kurkcuoglu, Pemra Doruker

**Affiliations:** 1 Department of Chemical Engineering and Polymer Research Center, Bogazici University, Istanbul, Turkey; 2 Department of Chemical Engineering, Istanbul Technical University, Istanbul, Turkey; University of Minnesota Twin Cities, UNITED STATES

## Abstract

The chaperone trigger factor (TF) binds to the ribosome exit tunnel and helps cotranslational folding of nascent chains (NC) in bacterial cells and chloroplasts. In this study, we aim to investigate the functional dynamics of fully-atomistic apo TF and its complex with 50S. As TF accomodates a high percentage of charged residues on its surface, the effect of ionic strength on TF dynamics is assessed here by performing five independent molecular dynamics (MD) simulations (total of 1.3 micro-second duration) at 29 mM and 150 mM ionic strengths. At both concentrations, TF exhibits high inter- and intra-domain flexibility related to its binding (BD), core (CD), and head (HD) domains. Even though large oscillations in gyration radius exist during each run, we do not detect the so-called ‘fully collapsed’ state with both HD and BD collapsed upon the core. In fact, the extended conformers with relatively open HD and BD are highly populated at the physiological concentration of 150 mM. More importantly, extended TF snapshots stand out in terms of favorable docking onto the 50S subunit. Elastic network modeling (ENM) indicates significant changes in TF’s functional dynamics and domain decomposition depending on its conformation and positioning on the 50S. The most dominant slow motions are the lateral sweeping and vertical opening/closing of HD relative to 50S. Finally, our ENM-based efficient technique -ClustENM- is used to sample atomistic conformers starting with an extended TF-50S complex. Specifically, BD and CD motions are restricted near the tunnel exit, while HD is highly mobile. The atomistic conformers generated without an NC are in agreement with the cryo-EM maps available for TF-ribosome-NC complex.

## Introduction

Trigger factor is the only ribosome-associated chaperone in bacteria that interacts with the growing chains during translation. This ATP-independent chaperone binds to the exit tunnel of ribosome [1–5] and facilitates the folding of newly synthesized proteins that emerge from this tunnel [6,7] by confining emerging chains [8,9]. TF acts as an unfoldase and a holdase, and thus prevents aggregation and misfolding of proteins [10]. Moreover, TF promotes the correct folding of proteins by protecting locally formed native contacts from distant interactions along the polypeptide so that misfolded states are avoided [11].

**Fig 1:**
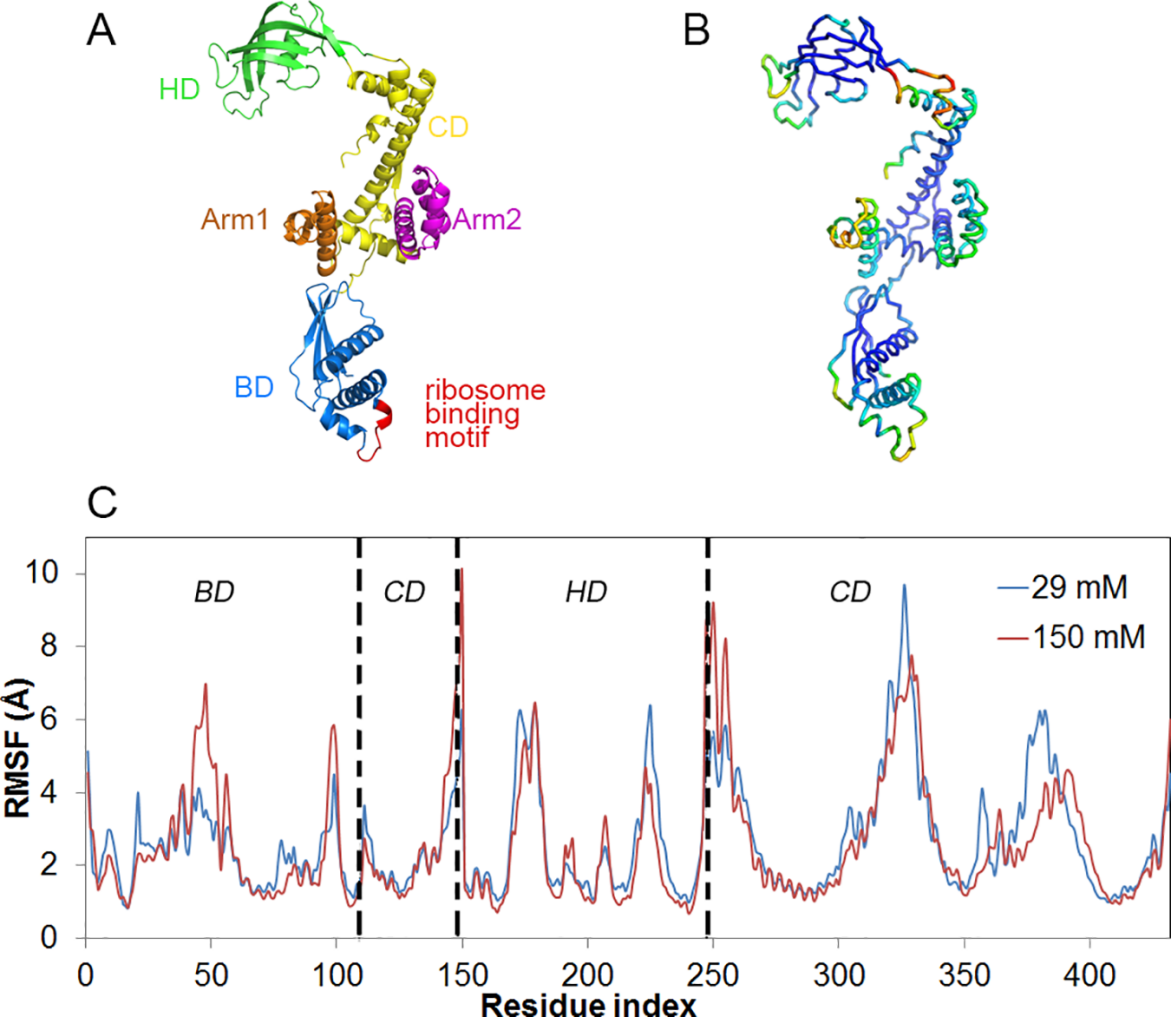
Structure and dynamics of apo TF in solution. (A) Domains of apo TF and its structural components with head (HD), core (CD) and binding domains (BD), (B) color-coded residue RMSF values sorted from highest (red) to lowest (blue) and (C) Residue RMSF based on domain-wise alignments, for 29 mM and 150 mM ionic strengths. Each domain is distinguished with dashed lines.

Findings on TF’s structure and function have been reviewed in detail [9,12], based on which its structural features can be summarized as follows. *E*.*coli* TF is a 432 residue long protein, composed of three domains (Fig 1A showing PDB ID: 1W26 [2]) [13]. Its ribosome-binding domain (BD) is the N-terminal domain (Met1- Glu110) forming the tail of this ‘dragon-shaped’ structure [2]. BD carries a signature motif (GFRxGxxP) that promotes TF’s specific binding to the L23 ribosomal protein located at the tunnel exit. At the other end of the structure lies the head domain (HD, Thr150-Pro246) with peptidyl prolyl isomerase activity that is not essential for TF’s function in co-translational folding [14]. The C-terminal domain (CD) forming the core of TF is composed of two non-contiguous domains (residues 111–149 and 247–432) and two arms, namely Arm1 (Ala304- Val346) and Arm2 (Asn358- Ala410).

BD and CD form an open cavity like a cradle, which accommodates hydrophobic residues [2,5,10,15] that present multiple binding sites for the nascent chain. At the same time, a high percentage of charged residues populate its surface, which may also act as contact sites, including a positive patch at the tip of BD that promotes ribosome binding. Located at the other end, HD is proposed to prolong the residence time of the protein within the cavity [2], and at the same time to promote NC binding.

The interaction cycle of TF with ribosome-NC complexes has been investigated by fluorescence spectroscopy [15] elucidating the following details. TF binds to the ribosome with a mean residence time of 10 s, regardless of whether there is an on-going translation process. Concurrently, TF undergoes a conformational expansion as it binds to the ribosome in monomeric form. After dissociating from the ribosome, TF-NC complex may stay intact for a residence time of more than 30 s, which is correlated with the hydrophobicity of the emerging chain. Due to excess concentration of TF, most of the ribosomes, including non-translating ones, are associated with monomeric TF, while a monomer-dimer equilibrium exists for unbound TF [16].

A cryo-EM and MD study [17] has shown that the degree of flexibility of TF bound to the ribosome decreases as the length of the nascent chain increases. Another coarse-grained MD study has shown that TF may slow down co-translational folding of a nascent chain by kinetic trapping of its unfolded ensemble [18]. Dynamics of apo TF have been studied by independent MD simulations performed at 50 and 150 mM ionic strengths [19,20], respectively. Both MD studies have indicated high inter-domain flexibility but at the same time pointed to the formation and stability of semi- and fully-collapsed states of TF, where either one or both of HD and BD are collapsed upon the core. The functional implications of such collapsed states are not clear, since experimental studies have so far emphasized the extended state acting as a cradle over the tunnel exit.

Our first aim in this work is to investigate the relevance of such semi or fully-collapsed states in terms TF-50S complex formation. As charged residues comprise more than 50% of TF’s solvent exposed surface area, we first aimed to investigate the effect of ionic strength on apo TF conformational dynamics using MD simulations. At 150mM ionic strength, TF populates more extended conformers in comparison to the crystal apo structure. Interestingly, in our simulations carried out with the CHARMM22 force field, which is different from previous work [19,20], the fully-collapsed state is not observed at all. Twelve distinct TF snapshots taken from 150mM runs were later docked onto the 50S, as a result of which the relatively extended states of TF were found more plausible for ribosome binding. We then assessed these complexes based on their positioning and conformation on 50S, which also dictated their collective modes obtained from ENM [21–23]. The variability of dynamics resulting from the diversity of docked TF conformations has not been considered in previous works. Finally, we applied a recently-developed, unbiased methodology named ClustENM [24] on a 50S-extended TF complex to visualize the intrinsically accessible states. As a result, 17 ribosome-TF complex conformers were obtained in conformity with the available cryo-EM structures [5,17].

## Materials and methods

### MD simulation protocols for apo TF

Five independent MD simulations that sum up to a total duration of more than one microsecond were performed using the NAMD Software Version 2.8. [25]. As the initial structure of apo TF molecule, chain A of the 2.70 Å resolution crystal structure (PDB ID: 1W26 [2]) of *E*. *coli* TF was chosen in all the simulations. The protein was solvated by creating a 12 Å water layer thickness in each direction by adding TIP3P type water molecules [26].

The ionic strength was set to 29 mM in two of the independent runs (200 ns and 500 ns long) with the addition of 23 Na^+^ ions for neutralization. In the remaining three runs (all 200 ns long), 133 Na^+^ and 110 Cl^−^ions were added to attain 150 mM that falls within the physiological conditions of bacterial cell. The solvated structures were then relaxed for 10,000 steps using conjugate gradient.

CHARMM22 force field was used in all simulations [27,28]. The dielectric constant of the system was taken as 1.0 as the default value for electrostatic interactions. The local interaction distance common to both Coulombic and van der Waals calculations were treated with a cut-off radius of 12 Å. The long-range electrostatic interactions were handled using the particle-mesh Ewald (PME) method [29] with a grid spacing of 2.0 Å.

All simulations were performed at a constant temperature of 310 K and pressure of 1 atm, resulting in the *NPT* ensemble. The Langevin piston Nosé-Hoover method was used to control fluctuations in the barostat [30,31]. The Langevin coupling coefficient for temperature was set to 50 ps^-1^. RATTLE [32] algorithm was used with 2 fs integration time step. The resulting coordinates were recorded at intervals of 4 ps.

### Elastic network model

The collective functional modes and dynamic domains in the apo TF and TF-50S complexes were analyzed via ENM [21,22]. The nodes that form the elastic network [21,33] were placed at the phosphate atoms for nucleotides and alpha-carbon atoms for amino acids. A cutoff distance of 13 Å was set for forming the elastic network of interactions. The first 10 harmonic modes were calculated using BLZPACK [34]. The cross-correlations based on these slow modes were analyzed to reveal the dynamic domains. The cross-correlation between the displacement vectors (Δ**R**) of residues *i* and *j* in the *k*^*th*^ mode is given as,

$$C_{ij}(k)=\frac{\Delta R_{i}(k)\cdot \Delta R_{j}(k)}{\left|\Delta R_{i}(k)\right|\left|\Delta R_{j}(k)\right|}$$ (1)

The correlations can be averaged over *k* normal modes by weighting with eigenvalues λ of normal modes as,

$$\langle C_{ij}\rangle =\frac{\sum_{k}C_{ij}\left(k\right)/\lambda_{k}}{\sum_{k}1/\lambda_{k}}$$ (2)

### Docking of TF onto the ribosome

Currently, an atomic structure for ribosome-TF complex is not available in the Protein Data Bank [35]. To construct the structure of TF-50S complex at atomic resolution, we used two crystal structures of 50S ribosome, partially containing TF binding domain with varying sizes. First structure is from *Haloarcula Marismortui* (PDB ID: 1W2B [2]), which has whole 50S unit together with ribosomal proteins; and this structure has only 35 residues of the TF binding domain. Second structure is from *Deinococcus Radiodurans* (PDB ID: 2AAR [3]), which carries a coarse-grained but a larger part of the binding domain (113 Cα atoms) on 50S ribosome, however this structure lacks most of the ribosomal proteins (only L23 and L29 are present). To obtain a 50S ribosome structure with all ribosomal proteins and maximum number of TF domain residues, we aligned 2AAR and 1W2B structures on top of each other using PyMOL [36]. Then, we used several alternative TF conformers from our MD runs at 150 mM ionic strength. For each conformer, we selected its BD and aligned this region on top of 113 Cα atoms-binding domain of TF from 2AAR. At the end, we obtained 12 different atomistic 1W2B-TF complexes, which have 7023 residues, corresponding 156,991 atoms in total. The formed complexes are listed as C_1 to C_12 in Table 1.

**Table 1:** Details of conformers docked to 50S.

Conformer	*R*_*g*_ (Å)	BD-Arm2^*a*^ (Å)	HD-Arm1^*b*^ (Å)	50S-Arm2^*c*^ (Å)	Positioning ^*d*^
C_1	35.0	42.3	45.1	7.5	Extended
C_2	37.4	42.8	60.1	11.3	Extended
C_3	35.8	44.7	56.1	9.5	Extended
C_4	38.0	46.1	66.0	12.0	Extended
C_5	35.5	46.4	48.8	21.5	Extended
C_6	33.9	48.0	44.4	17.4	Extended/ HD close contact
C_7	30.5	49.0	27.0	22.9	HD minor clash
C_8	31.0	46.7	30.5	14.2	HD clash
C_9	33.2	31.1	35.2	-	Arm2 clash
C_10	33.6	34.7	41.6	-	Arm2 clash
C_11	32.4	34.9	31.0	-	Arm2 clash
C_12	35.2	33.6	52.0	-	Arm2 clash

^*a*^ Distance between the centroids of BD and Arm2.

^*b*^ Distance between the centroids of HD and Arm1.

^*c*^ Distance between E386 (backbone) on the tip of Arm2 and Q102 (backbone) in chain L22 of 50S.

^*d*^ In extended conformers C_1 to C_4, both arms are positioned close to 50S, whereas only Arm1 lies close to 50S in C_5. In other conformers, there are minor or significant number of clashes between TF and 50S.

### Energy minimization using implicit solvent

The complexes without steric clashes between TF and 50S were subjected to energy minimization using implicit solvent in AMBER12 with ff10 force field parameters [37,38]. Pairwise generalized Born model [39,40] is used with a 16 Å cutoff for non-bonded interactions. Using modified generalized Born theory based on the Debye-Huckel limiting law for ion screening of interactions [41], the concentration of 1–1 mobile counter-ions in solution set to 0.1 M. Steepest descent is applied for 500 steps followed by conjugate gradient with convergence criterion of 0.01 kcal/mol/Å for energy gradient.

### ClustENM for atomistic TF-50S conformer generation

We employed our recently developed, iterative and unbiased method ClustENM [24,42] on one TF-50S structure to explore the conformational states intrinsically accessible to the complex, without the presence of polypeptide chain exiting the ribosomal tunnel. The complex used as a starting point corresponds to the docked minimized crystal structure (PDB ID: 1W26 [2], C_1 in Table 1) on 50S, where TF is in an extended conformation. Global modes were extracted from ENM [21] and the structure was deformed along the linear combination of first five modes using a deformation root mean square distance (RMSD) of 2 Å. Resulting conformers were clustered with an RMSD cutoff of 2 Å. A representative from each cluster except the one containing the initial structure was selected and subjected to energy minimization again. The whole procedure was repeated for the representatives, which now become parents of the next generation. We applied ClustENM for two generations, at the end of which 17 energetically minimized conformers at atomic resolution were obtained.

### Overview of the combined approach

In order to obtain realistic TF-ribosome complexes and to investigate their functional dynamics at time scales beyond the reach of full-atom MD simulations, we applied a combination of coarse-grained and atomistic techniques summarized below.

1. We employed full-atom MD simulations on apo TF in explicit solvent under physiological conditions. Representative MD conformers were chosen based on inter-domain distances that describe TF’s flexibility.
2. We docked representative conformers from Step 1 onto an existing crystal structure of partial-TF (binding region) and ribosome complex. This step produced possible binding complexes of full-length TF with ribosome, among which those without steric clashes were used in the next step.
3. ENM was performed on each complex to obtain collective, harmonic motions, as a result of which the functional dynamics of various coarse-grained conformers could be categorized.
4. Choosing one of the commonly observed complexes in Step 3, ClustENM was used to obtain full-atomistic conformations that may be detected in time scales of micro-seconds and above. The sampled conformations are then compared with EM maps to further assess their plausibility.

## Results

### Conformational dynamics of apo TF at two different ionic strengths

#### Inter and intra-domain flexibility

In order to observe the equilibration period for each independent run, RMSD calculations were carried out based on the backbone N-Cα-C atoms of TF. First, all snapshots of a specific run were aligned onto the initial snapshot using the whole TF molecule. The resulting RMSD between each snapshot and the initial structure was shown in S1 Fig for two of the runs. The fluctuations and RMSD values are extremely large for all runs, going up to 14–16 Å. To observe whether this is a consequence of inter-domain flexibility of TF as shown in previous studies [19,20], domain-wise alignments were carried out separately for BD, CD and HD. Domain-wise RMSDs are appreciably reduced (down to 6 Å in S1 Fig) but still point to significant intra-domain flexibility. Based on these RMSD plots, an equilibration period of 20 ns was discarded from each run in following analyses.

Residue root mean square fluctuations (RMSF) were calculated based on domain-wise alignments. Fig 1C shows the residue mobility in each domain and thus excludes inter-domain rearrangements of the whole TF. The fluctuations were calculated with respect to the average conformation of each domain determined by using all available snapshots for a specific ionic strength (two 29mM or three 150mM runs). Peaks are observed in similar regions for both ionic strengths with some variations in magnitude. Each domain is indicated on the figure by dashed lines, where CD is composed of two non-contiguous regions. Signature motif that promotes TF binding to ribosome gives the highest peak in the BD region at 150 mM ionic strength. Major peaks of HD correspond to two of its three flexible loops, one of which constitutes a binding region for the nascent alkaline phosphatase [10]. Lastly, two major peaks in the core domain correspond to its protruding arms, where the nascent alkaline phosphatase also binds to the second arm [10]. RMSF values color-coded by using the B-factor column in PyMOL tube representation are provided in Fig 1B for 150 mM ionic strength. The dark blue regions, mostly composed of helices, form the relatively rigid backbone of TF and the remaining parts are composed of flexible loops and inter-domain linkers. The residues linking CD to HD form a highly mobile and flexible neck.

#### More extended conformers at 150mM ionic strength

Radius of gyration (*R*_*g*_), which is an indicator of a chain’s overall size and compactness, is calculated for each snapshot.

$$R_{g}^{2}=\frac{1}{N}\overset{N}{\underset{k=1}{\sum }}{\left(R_{k}-R_{com}\right)}^{2}$$ (3)

**Fig 2:**
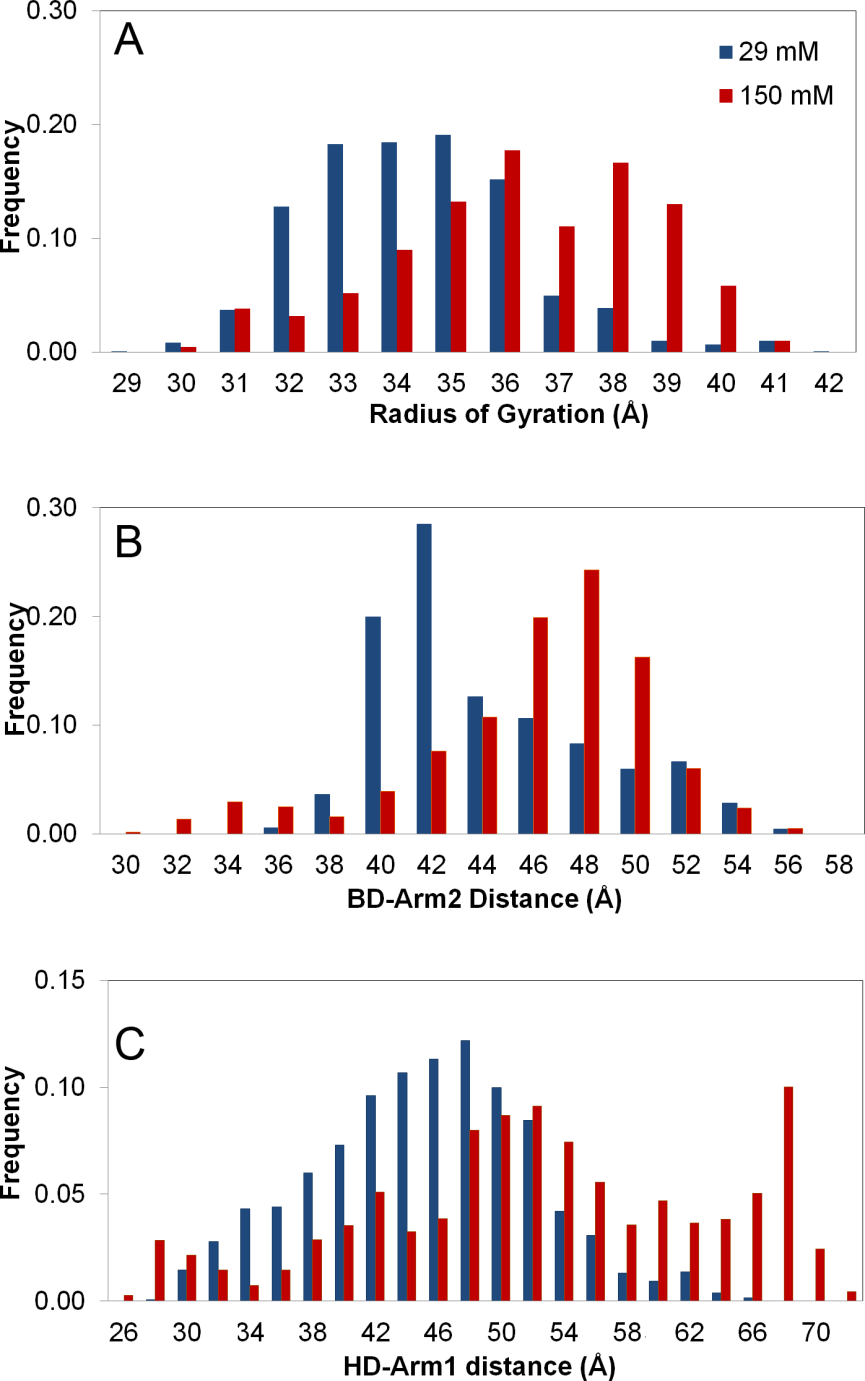
Histograms for radius of gyration and inter-domain distances. (A) Radius of gyration, (B) BD-Arm2 distance, and (C) HD-Arm1 distance for the 29mM (blue) and 150mM (red) runs. All distances cover broad ranges, in conformity with high flexibility of TF. 150 mM snaphots exhibit relatively more extended conformations.

Here *N* is the total number of residues, and **R**_*com*_ is the center of mass based on TF’s alpha-carbon atom coordinates. Normalized distributions/histograms of *R*_*g*_ values are presented for the recorded snapshots at different ionic strengths in Fig 2A. The highly populated *R*_*g*_ values fall in the range of 32–36 Å for 29 mM ionic strength. In contrast, more extended conformers (34–39 Å) are populated at the physiological ionic strength of 150 mM, possibly due to the large number of charged residues at the surface. The *R*_*g*_ of the minimized crystal structure is 35 Å, which is the starting point of our simulations (conformer C_1 in Table 1). In general, *R*_*g*_ profiles as a function of time exhibit an oscillatory behavior indicative of opening/closing motion of the HD and BD with respect to the CD (not shown). As TF is known to act as a cage-like protective tent for the newly synthesized nascent chains [43], more extended conformers will be discussed to be relevant for ribosome binding.

Fig 2B and 2C display histograms of BD-Arm1 and HD-Arm2 distances, which are calculated between the centroids of the specific domain pairs. These distances would directly affect the *R*_*g*_ values of the snapshots. The broad range of distances spanned is in conformity with the high inter-domain flexibility of TF. In comparison to respective HD-Arm1 and BD-Arm2 distances of 45 and 42 Å in the crystal structure, more extended states are populated especially at 150 mM ionic strength.

**Fig 3:**
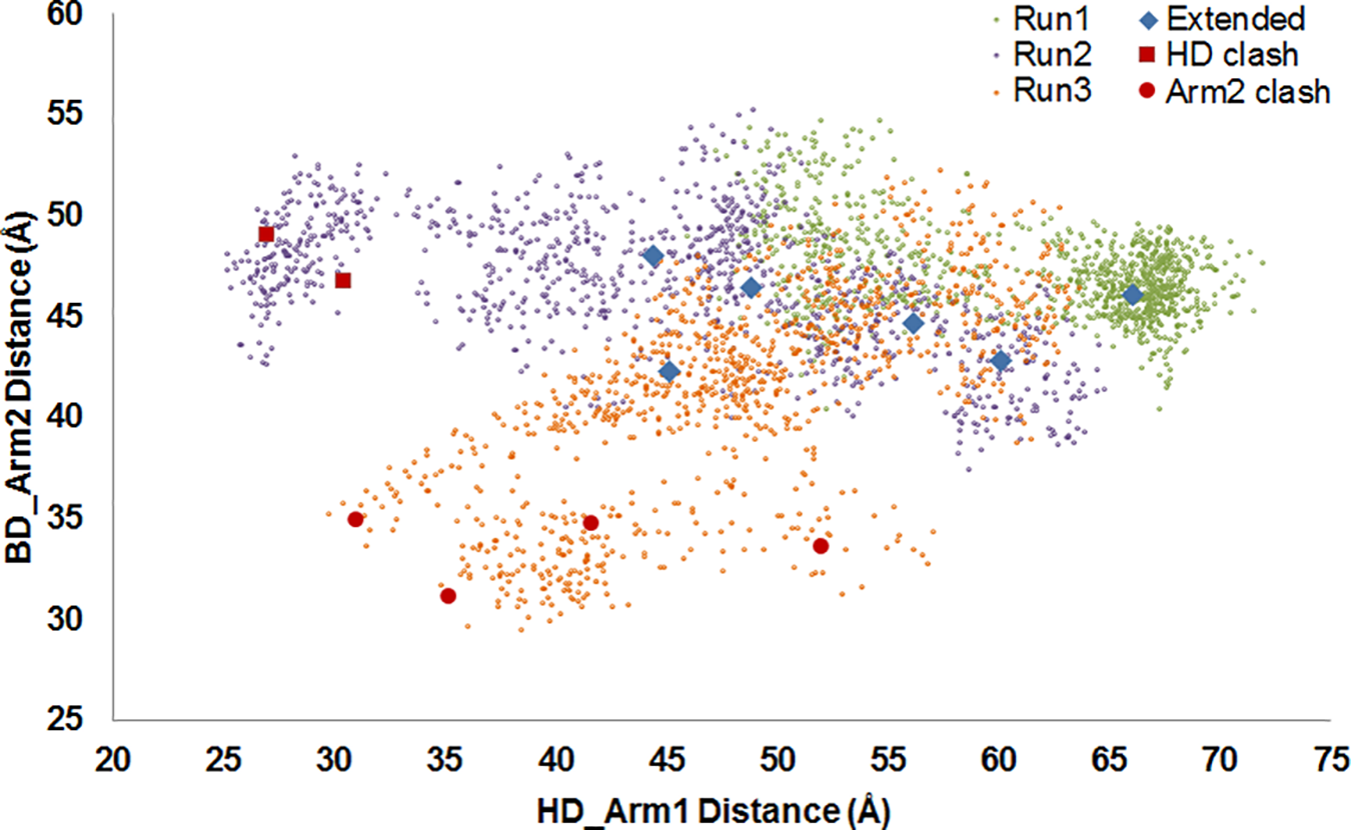
HD-Arm1 distance *vs*. BD-Arm2 distance plot for three independent runs at 150 mM ionic strength. Blue diamonds correspond to extended conformers docked onto 50S without clashes. Red squares and spheres are docked conformers with specific clashes, as listed in Table 1.

To further indicate the diversity of visited conformational states, HD-Arm1 vs. BD-Arm2 distances are plotted in Fig 3 for three independent 150 mM runs (S2 Fig for two 29 mM runs). The distributions are noted to be broader at the higher ionic strength, indicating that both semi-collapsed (HD closed onto the core) and extended states are visited without getting stuck in neither of these states. In short, TF is sensitive to ion concentration. The dominance of relatively extended states at 150 mM strength may enhance ribosome binding, as will be discussed in the context of TF-50S complexes.

#### Collapsed states are not visited

Recently, two MD studies have focused on conformational dynamics of apo TF in explicit solvent [19,20]. The former study [19], reported 12 independent 250 ns long runs at 50 mM ionic strength and 295 K. In most runs performed with AMBER03 [44], a so-called ‘fully-collapsed’ state (collapse of HD and BD onto the core) was observed with the HD-Arm1 and BD-Arm2 distances less than 26 and 20 Å, respectively. Such a state is not noted in our simulations (see Fig 3 and S2 Fig for details). In the rest of the simulations with OPLS-AA [45], a semi-collapsed state (with collapsed HD only) was reported, which is observed in a limited number of snapshots in one of our 150 mM runs. In the latter study [20], two 1.5 μs long MD runs have been performed with OPLS-AA/L [46] and AMBER ff99SB-ILDN [47,48] force field parameters at 298 K. Early in the OPLS simulation, TF adopts a highly compact (‘collapsed’) state with an *R*_*g*_ around 26 Å, not visited in our simulations.

The fact that such fully-collapsed and quite stable states are not observed in our runs at two different ionic strengths may result from differences in force fields and/or simulation conditions employed. Specifically, we used the CHARMM22 force field and the physiological temperature of 310 K, both of which are different from previous studies.

TF’s function as a chaperone necessitates its proper binding to the exit tunnel of the bacterial ribosome. In view of partially or fully collapsed conformations, the HD will be in too close proximity to the ribosome surface. As a result, the extension of nascent chain within TF would be hindered as well as its specific interactions with TF, which have been reported by previous experimentation [5,10,49]. Therefore, relatively more extended conformations, observed in our simulations, seem to be more plausible in terms of the ribosome-binding function of TF. Next we will discuss the dynamics of several TF conformations docked to the 50S.

### Conformational dynamics of TF- 50S complex

#### Comparison of apo and 50S-bound dynamics

We wanted to observe the changes in collective dynamics upon binding to 50S. First, we formed several TF-50S complexes by docking diverse apo TF conformers from 150 mM runs onto the 50S, as described in Methods. For this procedure, 12 apo conformers were selected from distinct regions on the BD-Arm2 vs. HD-Arm1 plot (Fig 3 for 150 mM runs), which are listed in Table 1 and explicitly indicated on Fig 3. These TF-50S complexes are shown in S3 Fig, where the docked conformer C_1 corresponds to the minimized TF crystal structure (1W26, chain A).

**Fig 4:**
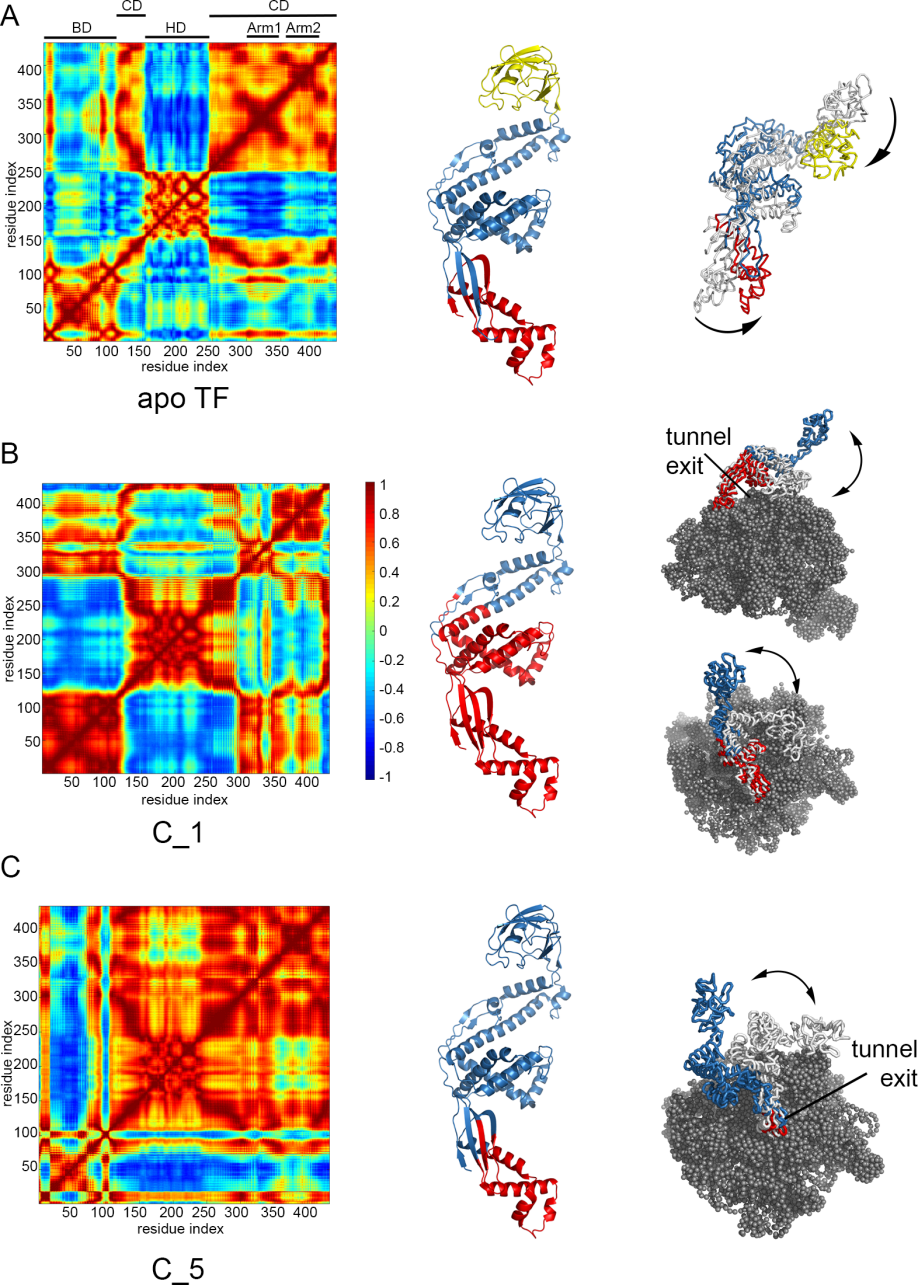
Residue cross-correlations and harmonic motions for TF structures. In the left panels, residue cross-correlations are displayed for TF structures in (A) its apo form and (B), (C) two different complexes with 50S, based on 10 slowest ENM modes. Blocks on the maps point to domain decomposition on TF, as shown with different colors on cartoon representations in the middle panels. In the right panels, harmonic motions for the first modes are shown only for TF docked to 50S (gray spheres) from different perspectives, with the same domain coloring.

Residue cross-correlation matrices are calculated based on the cumulative action of the first 10 ENM modes (see Eqs 1 and 2) and shown in Fig 4 for apo TF, C_1 and C_5. The correlated blocks within TF can be more clearly observed in the first mode, as the slowest mode dictates/dominates the cumulative correlations in the slowest modes. Therefore, S4 and S5 Figs compile the first mode correlations of TF for 50S-complexes and apo conformers, respectively.

Slowest functional motions of TF in its apo form (Fig 4A and S5 Fig) point to three dynamic domains, distinctly corresponding to BD, HD and CD including the arms, as depicted from Fig 4A left and middle panels. Principal component analyses based on 150 mM runs were also carried out to reveal the collective dynamics of TF (not shown) [50,51]. Domain decompositions obtained from MD runs and ENM were found consistent, indicating an anti-correlated motion between these three major domains.

In contrast to apo TF dynamics, collective motions of C_1 were rather restricted due to the large number of interactions between BD and ribosome at the tunnel exit, as well as the close contact of the two arms with the 50S. Such positioning of TF on 50S led to two dynamic domains dividing the TF structure from its middle as shown in Fig 4B. These domains comprised BD and arms, and upper portion of CD and HD.

We further investigated other TF-50S complexes, which are explicitly shown on Fig 3 and listed in Table 1. Relative positioning of TF with respect to the large subunit was similar for several structures as seen in S3 Fig, although their BD-Arm2 and HD-Arm1 distances were different. Among these complexes, C_1 to C_6 do not have any steric clashes between TF and 50S, but the rest has minor or significant number of clashes, resulting from close contact of either HD or Arm2, which are reported in Table 1. Accordingly, ENM was applied to the first six complexes and additionally to C_7 including minor clash between HD and 50S.

The collective motions were clearly altered upon complex formation depending on the conformation and positioning of TF docked on 50S. For C_1 to C_4, the cross-correlations (S4 Fig) and hence the domain decompositions closely resemble each other. In all these complexes, both arms make close contact with 50S. In contrast, Arm2 in C_5 is positioned relatively more distant to 50S (see Table 1) and the domain decomposition is different. Here, HD and CD merge and are separated from the BD by a hinge region in Fig 4C. We additionally considered C_6, where HD lies quite close to 50S, and C_7 with minor steric clash between HD and 50S. In both of these complexes, TF moves as a fully correlated entity together with 50S, as depicted from residue cross-correlations of the first ENM mode (see S3 and S4 Figs). We did not investigate the other complexes with extensive structural overlaps between TF and 50S, namely C_8 with HD clash and C_9 to C_12 with Arm2 protruding into 50S.

When we focus on the slow mode deformations of the complexes (S6 and S7 Figs), lateral sweeping-like and opening/closing motions of TF relative to 50S stand out consisting of a single hinge. In contrast, TF in its apo form seems to merit only from two fixed hinge regions separating BD, CD and HD, as our MD and ENM analyses indicate (also see S5 Fig). In summary, our normal mode calculations suggest that according to its relative position and conformation on the ribosome, TF is able to modulate its flexibility using variable hinge regions that can alter between functional modes of motions (S4, S6 and S7 Figs and Fig 4). It should be noted that TF upon binding to the large subunit can inherently make such motions, even in the absence of a nascent chain, as is the case in our calculations.

#### Conformational sampling of the TF-50S complex

We further performed an atomistic conformational sampling of the complex based on slowest five modes. Our aim was to observe the extent of TF motions on the 50S using ClustENM, an efficient, unbiased ENM-based technique, which has proven successful in providing atomistic sampling for a variety of highly flexible proteins and the 70S ribosome [24,42].

**Fig 5:**
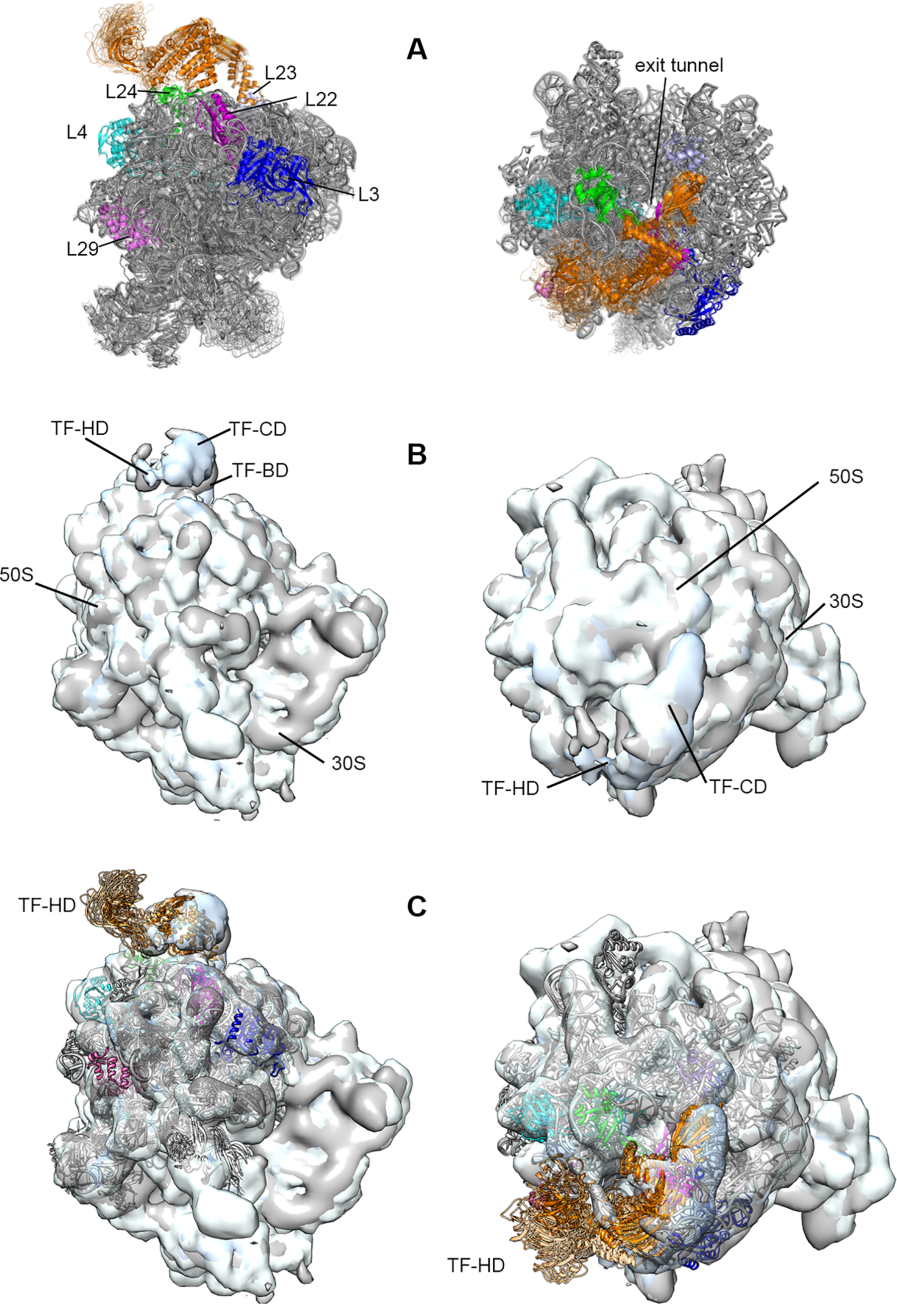
Atomistic TF-50S conformers from ClustENM fitted into cryo-EM maps. (A) Starting structure (opaque) and generated conformers (transparent) of TF-50S complex from ClustENM shown for side and top views. TF (orange), 50S (grey) and several ribosomal proteins neighboring TF are explicitly shown. In the bound state on ribosome, CD and BD of TF are almost fixed, whereas HD is highly flexible. HD moves in parallel to the surface of ribosome but also experiences up-down motion (B) Side and top views for three aligned cryo-EM map densities (grey: EMD-1499, pink: EMD-2696, cyan: EMD-2711) using Chimera, revealing the lateral head domain motion of TF in the presence of nascent polypeptide chain. (C) Side and top views for all 17 atomic conformers from ClustENM fitted into superimposed cryo-EM maps in (B) showing good conformity between our ENM calculations and the experimental data.

We applied ClustENM on the extended TF-50S complex, namely C_1, to visualize its accessible states, which resulted in a total of 17 distinct conformers after performing two generations of ClustENM. The superimposition of TF-50S conformers from ClustENM reveals high mobility of the HD in Fig 5A and S1 Movie. The BD and the arms of CD remain relatively stable on ribosomal exit tunnel, which is contrary to their significant mobility observed in MD simulations of monomeric apo TF in solution [19,20,50,51]. This supports the view that upon binding on ribosome surface, BD and the arms provide a relatively stable, protected folding space for the nascent polypeptide chain [2]. This observation further agrees with the cryo-EM maps (EMD-1499 [5], EMD-2711 and 2696 [17] with respective resolutions of 19, 13 and 13 Å) of TF-ribosome complex in Fig 5B aligned in Chimera [52]. The head movement is apparent in this data, while BD and CD are stable on ribosome in the presence of nascent polypeptide. When our ClustENM conformers are fitted to these cryo-EM data, head movement takes place both in lateral (sweeping-like motion) and vertical directions (Fig 5C and S8 Fig). In fact, these HD motions on 50S is in accordance with the recent cryo-EM map and the related MD study, where the landing of HD onto ribosome surface was observed in presence of a nascent polypeptide chain [17]. The lateral and vertical motions can serve as to collect and guide the nascent chain’s progress and interactions within the confined space between TF and 50S. Furthermore, the nascent chain is known to interact with all domains of TF, including the HD [5,10].

We also calculated overlap values between the first five ENM eigenvectors (**u**_*ENM*_) based on starting conformer C_1 and the 17 displacement vectors (**v**_***conf***_) that indicate the deformations/movements from C_1 to each of the distinct ClustENM conformers. The goal here is to observe if ClustENM samples any TF conformations beyond the reach of the initial ENM modes alone. The overlap value, calculated from Eq (4), indicates how much each ENM mode is conserved, where 0 and 1 mean no and perfect overlap, respectively.

$$overlap=abs\left(\frac{u_{ENM}\cdot v_{conf}}{\left|u_{ENM}\right|\left|v_{conf}\right|}\right)$$ (4)

When the maximum overlap value among the first five ENM modes is chosen for each ClustENM conformer, the range falls between 0.48–0.86. Among 17 ClustENM conformers, 11 display an overlap below 0.7. Thus, ClustENM is indeed able to sample conformations beyond the reach of initial ENM modes alone due to linear mode combination and re-evaluation of ENM modes for distinct conformers at each generation.

We should stress that the TF-50S complexes in our study were generated without the nascent polypeptide, which indicates that HD flexibility is an intrinsic property of the complex even in the absence of the peptide. Our ENM underlies the importance of positioning of TF on its dynamics and coupling with the 50S. If CD-50S interactions are loose in the complex while BD remains fixed, TF is expected to move more independently and to sweep more space on the 50S surface. In our ClustENM conformer C_1, CD lies close to 50S, therefore more restricted conformers are generated, in good conformity with cryo-EM data.

## Concluding remarks

Five independent MD simulations were performed at two different ionic strengths (29 and 150 mM) in explicit solvent to investigate the conformational dynamics of apo TF. High inter-domain and intra-domain flexibility is evident, which seems to be crucial for its binding to ribosome and its chaperone activity. Conformational states of TF were further investigated based on the radius of gyration and inter-domain distances, namely those between HD-Arm1 and BD-Arm2. Opening/closing of HD onto the CD is commonly observed, while more extended structures are preferred at the physiological ionic strength of 150 mM. We should also state that full BD-closure on CD is not observed in our current simulations, which was highlighted in previous works [19,20] as leading to fully collapsed states of TF. Indeed, such fully-collapsed conformers would hinder proper binding of TF to ribosome and functioning of the complex during translation, as will be further discussed next.

Twelve distinct TF snapshots chosen from MD runs were later docked onto the 50S by alignment onto a fragment of BD that was co-crystallized with ribosome 50S subunit. Among these, relatively more extended conformers, i.e. blue diamonds located on the upper right quadrant of Fig 3, lead to more plausible alignments without any steric clashes between TF and 50S. Moreover, in such extended states, TF exposes its inner residues as well as its binding sites to the nascent chain coming out of the ribosome tunnel. In contrast, when HD and/or BD are collapsed onto CD with relatively short distances between HD-Arm1 and/or BD-Arm2, steric clashes between TF and 50S occur during docking. Indeed, this finding underlines the experimental statement that TF undergoes a conformational expansion as it binds to the ribosome in monomeric form [10]. Therefore, extended states of TF appear more favorable in terms of 50S binding.

ENM performed on the docked conformers without clashes leads to insights about some common features of global dynamics. In contrast to three distinct domains (HD, CD and BD) observed for apo TF conformers, the TF-50S complexes generally consist of a single hinge, which either divides the CD into half or is located between BD and CD (Fig 4) according to the positioning of TF. Additionally, in conformers showing close contact of HD with CD, the TF moves as a single entity, i.e. in a fully correlated manner with 50S. Thus, the relative positioning of TF and its domains with respect to the 50S affects its interactions, flexibility and dynamics to a great extent. Interestingly, TF can modify its flexibility using variable hinge locations related with its positioning on 50S.

We also applied our recently developed conformer generation technique ClustENM on 50S-extended TF complex to reveal the dynamics upon binding at atomic resolution. Generated conformers were in line with the cryo-EM data [5,17], where the BD and CD are relatively rigid as opposed to apo TF in solution, and the HD is highly flexible. As for dynamics, we pronouncedly observe the sweeping motion of HD parallel to ribosome surface and the vertical movement of HD relative to the ribosome surface in the absence of nascent polypeptide chain. At the same time, these intrinsic motions of flexible TF appear critical for accommodating nascent chains of various sizes and shapes.

## Supporting information

S1 Fig RMSD profiles of TF. (a) 29 mM and (b) 150 mM runs after overall (whole TF) and domain wise (BD, CD and HD) alignments onto the initial structure of each run.
(TIF)

S2 Fig HD-Arm1 distance *vs*. BD-Arm2 distance plot for two independent runs at 29mM ionic strength. (TIF)

S3 Fig Different conformations of TF (orange) obtained from clustering analysis, then docked to 50S structure (grey). Complexes are shown from two different perspectives.
(TIF)

S4 Fig Cross-correlation maps based on first ANM mode for distinct TF conformers docked on 50S as shown in S3 Fig. For clarity, cross-correlations for 50S residues are omitted.
(TIF)

S5 Fig Cross-correlation maps based on first ANM mode of selected apo TF conformers from 150mM runs. Three dynamic domains are observed in most of the relatively extended apo conformers (C_2, C_3, C_5) (same as C_1 in Fig 4A), corresponding to the structural domains, BD, CD and HD. For the relatively compact conformer C_7, a different picture emerges.
(TIF)

S6 Fig Four slowest mode deformations of C_1, which is further used in ClustENM calculations. Deformations of TF are shown in red and pink ribbons for each mode, and 50S is in grey spheres. To clearly visualize each harmonic mode, conformations are given from two different perspectives (side and top views). In the first mode, TF moves in a lateral direction sweeping the surface of 50S. In the second mode, an opening/closing motion of the HD is clearly observed in the side view. In the third mode, deformations of the L7/L12 stalk are dominant, while motions of TF seem to be restricted. The fourth mode also involves a lateral deformation of the TF. Hinges (blue dashed lines) are explicitly shown for the first two modes.
(TIF)

S7 Fig Four slowest mode deformations of C_5. Deformations of TF are shown in red and pink ribbons for each mode, and 50S is in grey spheres. To clearly visualize each harmonic mode, conformations are given from two different perspectives (side and top views). In the first mode, TF again moves in a lateral direction sweeping the surface of 50S. In the second mode, an opening/closing motion of the HD is clearly observed in the side view. The third mode also involves a lateral deformation of the TF. Hinges (blue dashed lines) in the first two modes are located closer to the BD, in comparison to C_1 in S6 Fig.
(TIF)

S8 Fig ClustENM TF conformers fitted on cryo-EM data. (a) EMD-1499 (grey), (b) EMD-2696 (light blue), and (c) EMD-2711 (white).
(TIF)

S1 Movie Atomistic TF-50S conformers generated by ClustENM. (AVI)
