# Supplementary figures and images for: Conformational dynamics of bacterial trigger factor in apo and ribosome-bound states
Source: PLoS One. 2017 Apr 24;12(4):e0176262. doi: 10.1371/journal.pone.0176262 (PMC5402958; doi:10.1371/journal.pone.0176262)

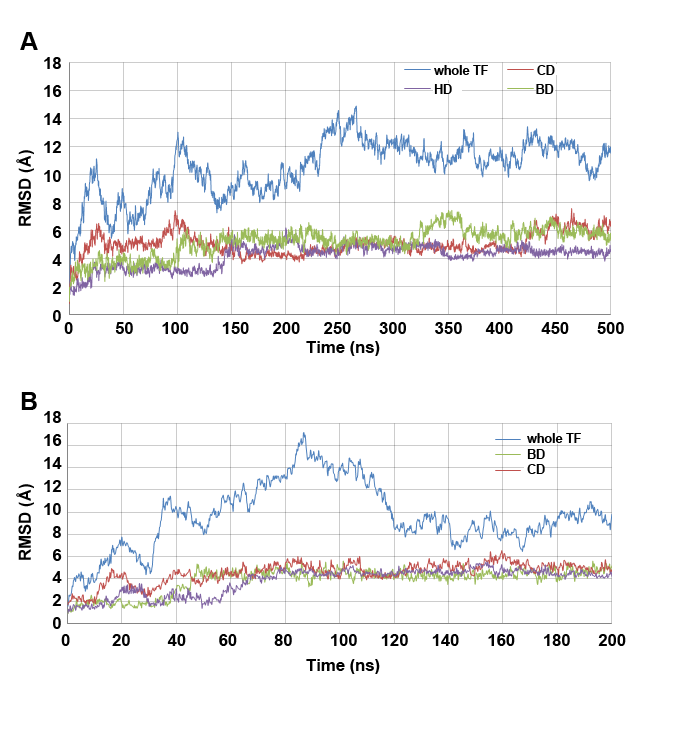

Supplement: S1 Fig — (a) 29 mM and (b) 150 mM runs after overall (whole TF) and domain wise (BD, CD and HD) alignments onto the initial structure of each run. (TIF) [file pone.0176262.s001.tif]

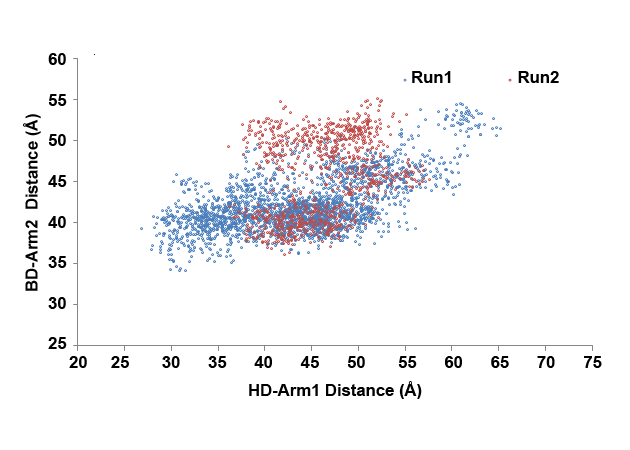

Supplement: S2 Fig — (TIF) [file pone.0176262.s002.tif]

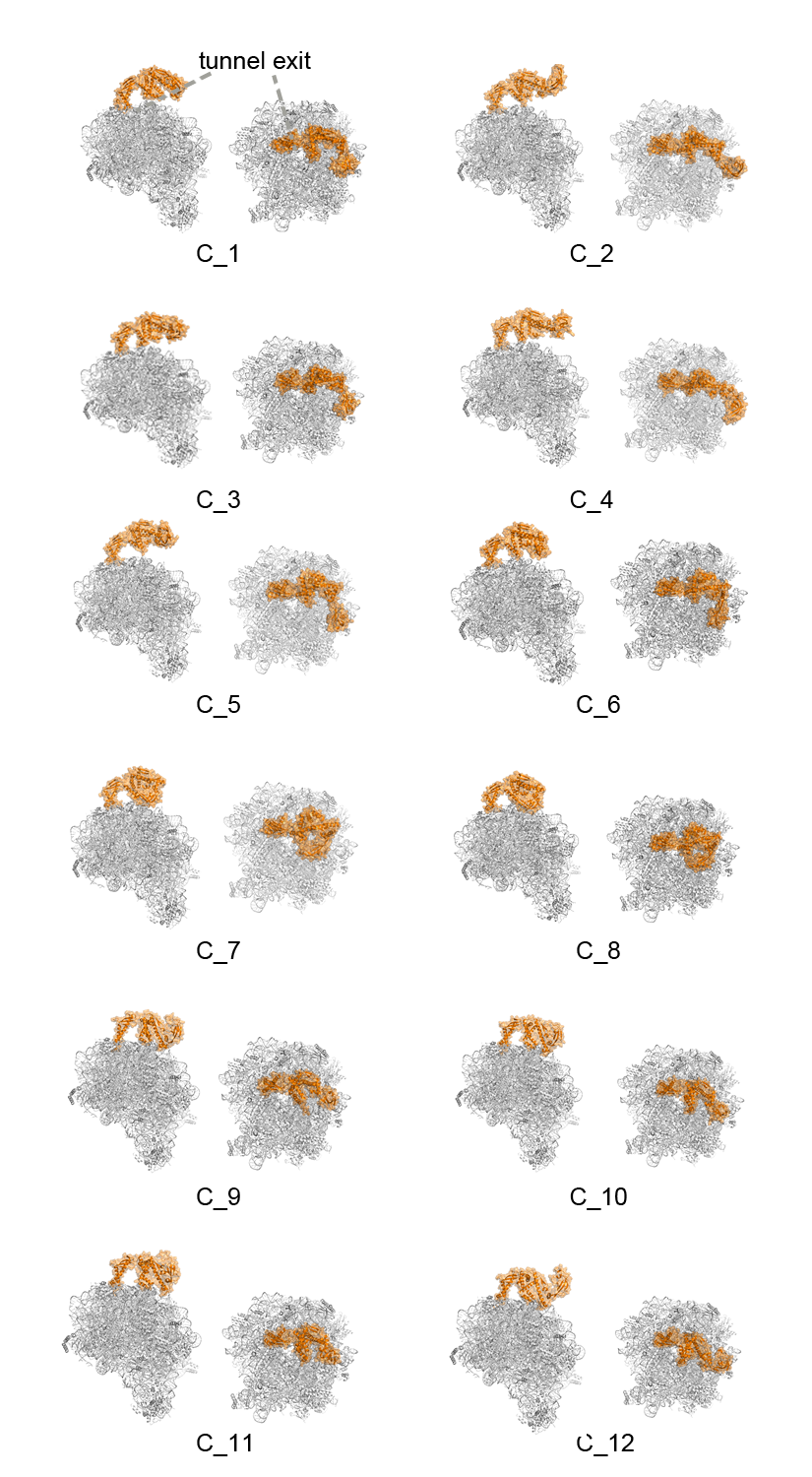

Supplement: S3 Fig — Complexes are shown from two different perspectives. (TIF) [file pone.0176262.s003.tif]

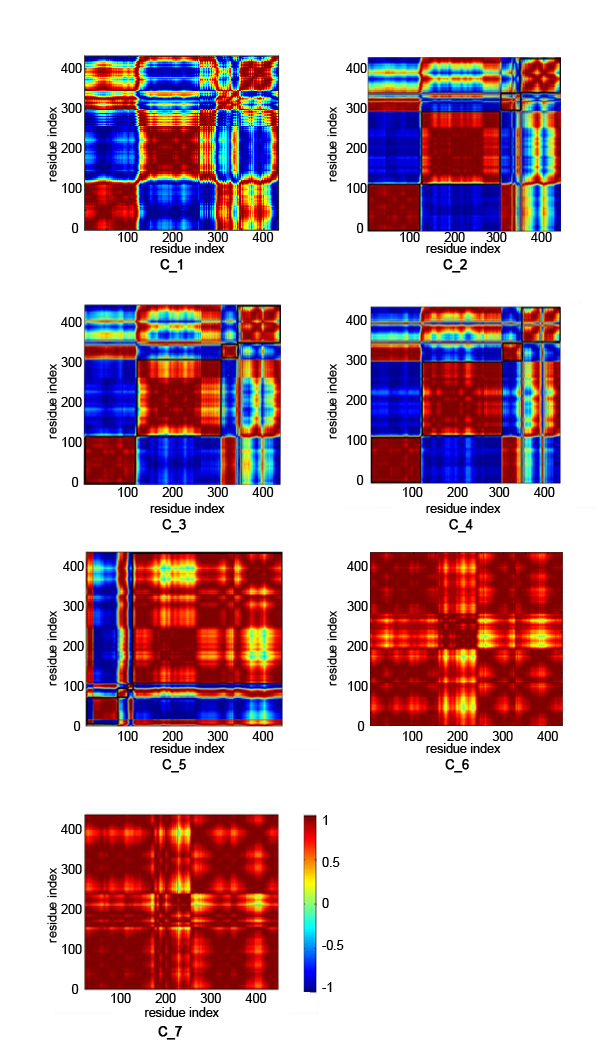

Supplement: S4 Fig — For clarity, cross-correlations for 50S residues are omitted. (TIF) [file pone.0176262.s004.tif]

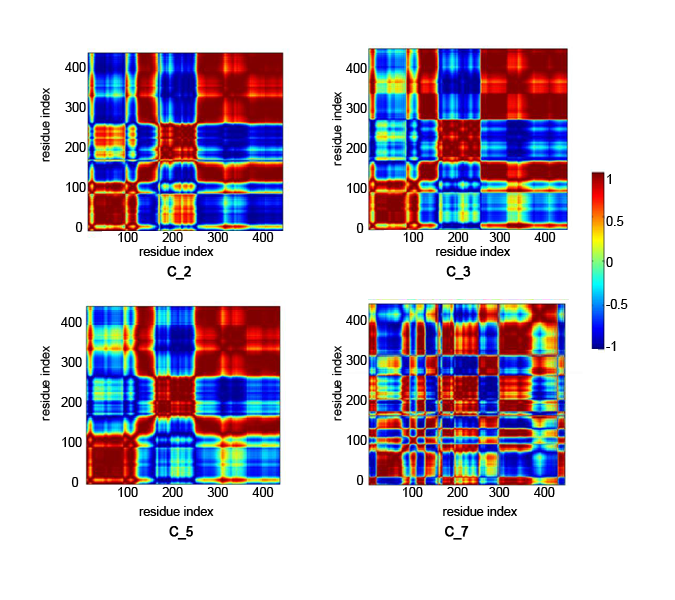

Supplement: S5 Fig — Three dynamic domains are observed in most of the relatively extended apo conformers (C_2, C_3, C_5) (same as C_1 in Fig 4A), corresponding to the structural domains, BD, CD and HD. For the relatively compact conformer C_7, a different picture emerges. (TIF) [file pone.0176262.s005.tif]

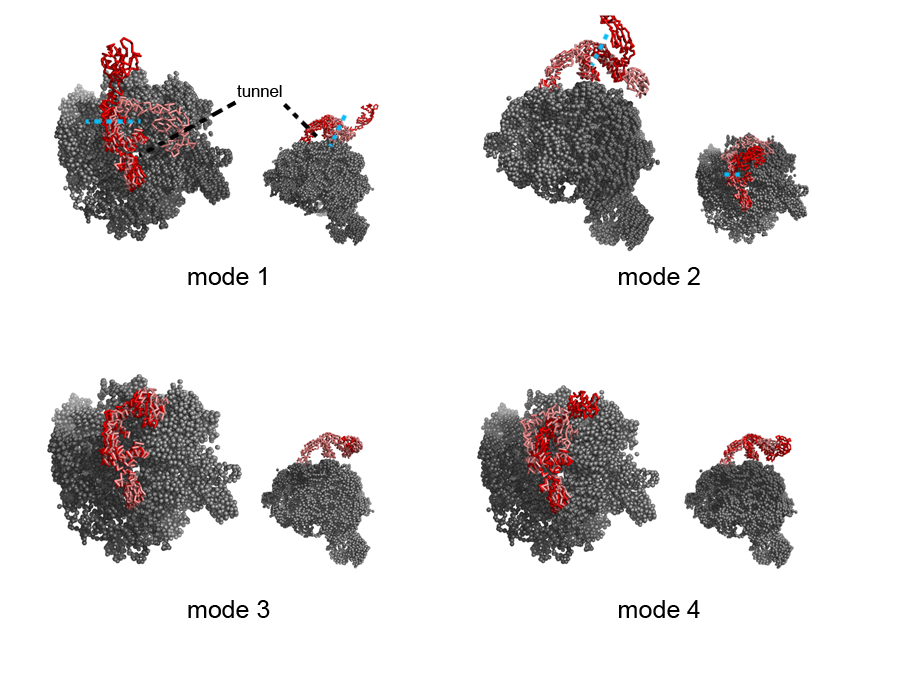

Supplement: S6 Fig — Deformations of TF are shown in red and pink ribbons for each mode, and 50S is in grey spheres. To clearly visualize each harmonic mode, conformations are given from two different perspectives (side and top views). In the first mode, TF moves in a lateral direction sweeping the surface of 50S. In the second mode, an opening/closing motion of the HD is clearly observed in the side view. In the third mode, deformations of the L7/L12 stalk are dominant, while motions of TF seem to be restricted. The fourth mode also involves a lateral deformation of the TF. Hinges (blue dashed lines) are explicitly shown for the first two modes. (TIF) [file pone.0176262.s006.tif]

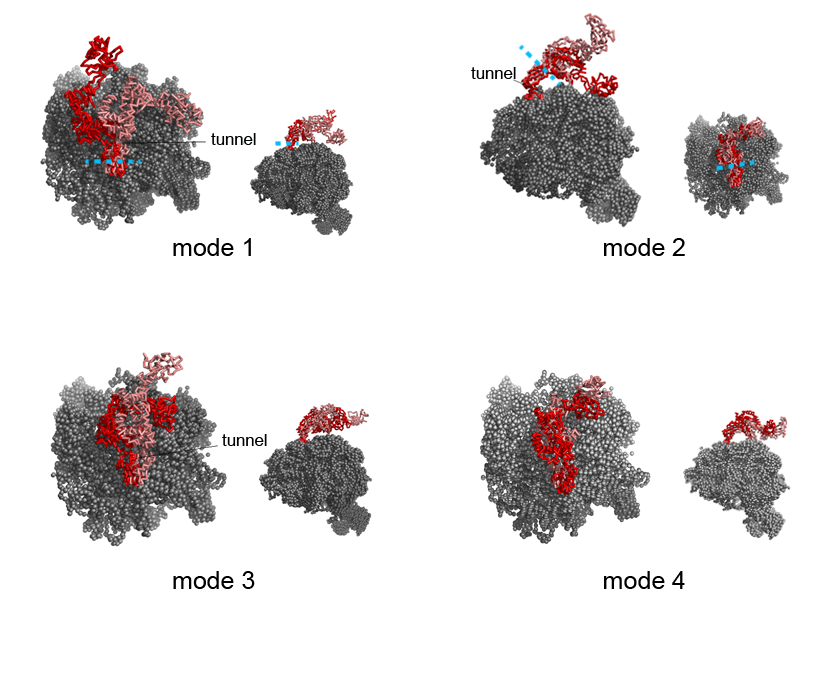

Supplement: S7 Fig — Deformations of TF are shown in red and pink ribbons for each mode, and 50S is in grey spheres. To clearly visualize each harmonic mode, conformations are given from two different perspectives (side and top views). In the first mode, TF again moves in a lateral direction sweeping the surface of 50S. In the second mode, an opening/closing motion of the HD is clearly observed in the side view. The third mode also involves a lateral deformation of the TF. Hinges (blue dashed lines) in the first two modes are located closer to the BD, in comparison to C_1 in S6 Fig. (TIF) [file pone.0176262.s007.tif]

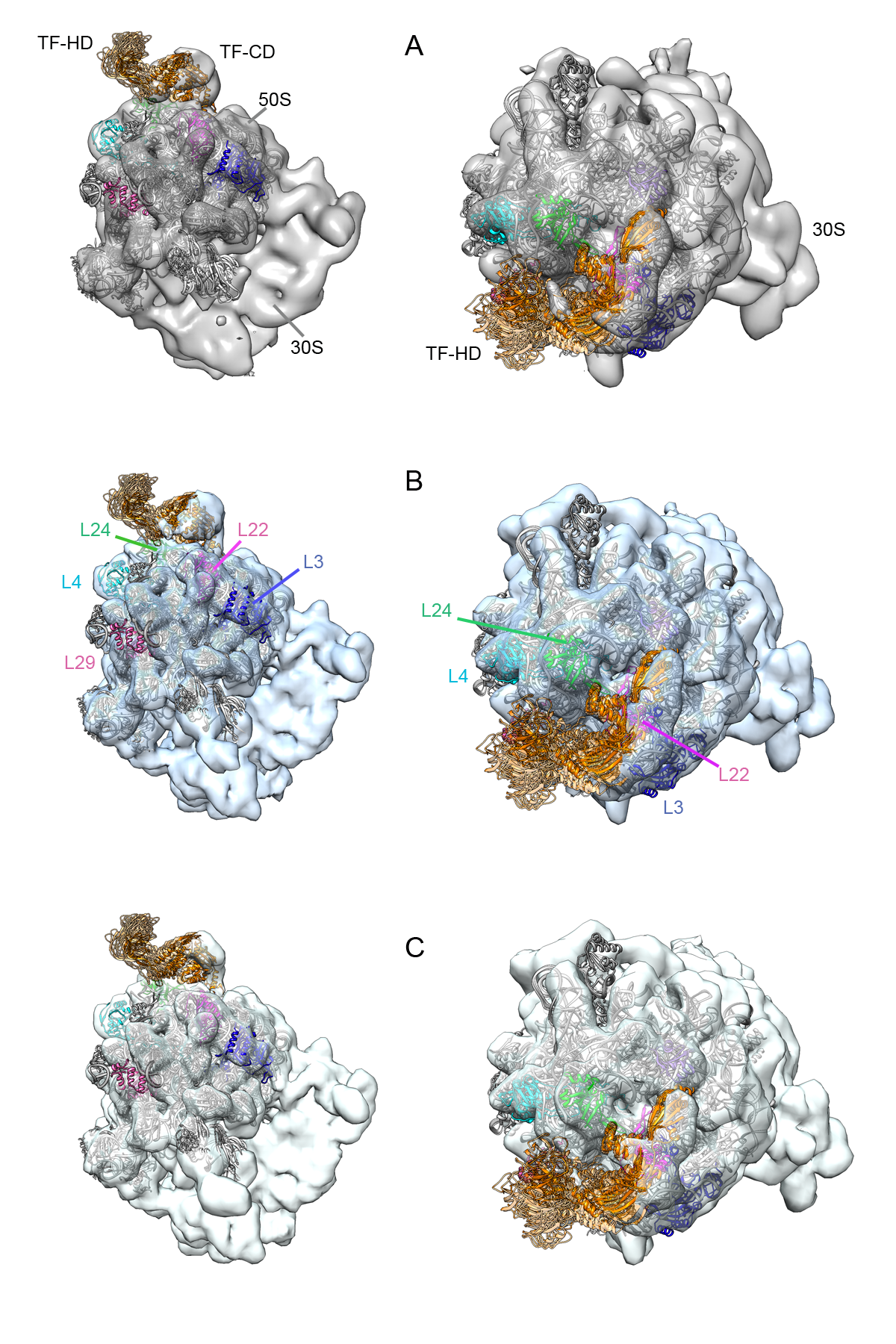

Supplement: S8 Fig — (a) EMD-1499 (grey), (b) EMD-2696 (light blue), and (c) EMD-2711 (white). (TIF) [file pone.0176262.s008.tif]
